# HIV incidence and risk factors among transgender women and cisgender men who have sex with men in two cities of China: a prospective cohort study

**DOI:** 10.1186/s40249-022-00947-3

**Published:** 2022-03-07

**Authors:** Duo Shan, Zhen Ning, Maohe Yu, Huang Zheng, Jie Yang, Hui Gong, Jian Li, Hui Liu, Lu Liu, Vania Wang, Xiong Ran, Mengjie Han, Dapeng Zhang

**Affiliations:** 1 National Center for AIDS/STD Control and Prevention, Chinese Center for Disease Control and Prevention, Beijing, 102206 People’s Republic of China; 2 Division of AIDS/STD Control and Prevention, Shanghai Municipal Center for Disease Control and Prevention, Shanghai, 200336 People’s Republic of China; 3 Division of AIDS Control and Prevention, Tianjin Centers for Disease Control and Prevention, Tianjin, 300011 People’s Republic of China; 4 Shanghai CSW&MSM Center, Shanghai, 200336 People’s Republic of China; 5 Shenlan Public Health Consulting Service Center in Tianjin, Tianjin, 300171 People’s Republic of China; 6 Department of Epidemiology and Health Statistics, School of Public Health, Tianjin Medical University, Tianjin, 300070 People’s Republic of China; 7 Department of Geography, University of California Santa Barbara, Santa Barbara, CA 93106 USA

**Keywords:** HIV, AIDS, Incidence, Men who have sex with men, Transgender women

## Abstract

**Background:**

HIV epidemic among men who have sex with men (MSM) remains a major public health concern in China. Despite a growing body of research on transgender women worldwide, little is known about Chinese transgender women within MSM. We sought to estimate HIV incidence and distinguish risk factors of HIV acquisition among them from that among cisgener (non-transgender) MSM (cis-MSM).

**Methods:**

We conducted an open cohort study among Chinese MSM, including those who were identified as transgender in Shanghai and Tianjin. Participants were initially recruited by local community-based organizations from January to June, 2016, and were followed up approximately every 6 months until June 2018. At each visit, a structured questionnaire was used to gather information on demographics, sexual risk behaviors, and HIV status. HIV incidence was calculated as the number of seroconversions divided by total number of person-years of follow-up among HIV-negatives at baseline. Risk factors of HIV acquisition were assessed by univariate and multivariate Cox regression models with time-dependent variables.

**Results:**

A total of 1056 participants contributed 1260.53 person-years (PYs) of follow-up, 33 HIV seroconversions occurred during the follow-up period, yielding an estimated HIV incidence of 2.62 (95% *CI* 1.80–3.68) per 100 PYs. HIV incidence among transgender women was 4.42 per 100 PYs, which was significantly higher than that of 1.35 per 100 PYs among cis-MSM, demonstrating a threefold higher odds of HIV infection than cis-MSM. For transgender women, those lived locally ≤ 2 years (adjusted hazard ratio [a*HR*] = 1.76, 95% *CI* 1.13–2.76) and unprotected anal sex last time (a*HR* = 4.22, 95% *CI* 1.82–9.79) were more likely to acquire HIV. For cis-MSM, factors associated with HIV acquisition were frequency of anal sex ≥ 3 times in past one month (a*HR* = 4.19, 95% *CI* 1.06–16.47) and unprotected anal sex last time (a*HR* = 5.33, 95% *CI* 1.52–18.73).

**Conclusions:**

Compared to cis-MSM, transgender women were at higher risk of HIV acquisition, highlighting an urgent need of tailored prevention. Future HIV program should consider to include them to ensure that this population in China are not left behind.

**Graphical abstract:**

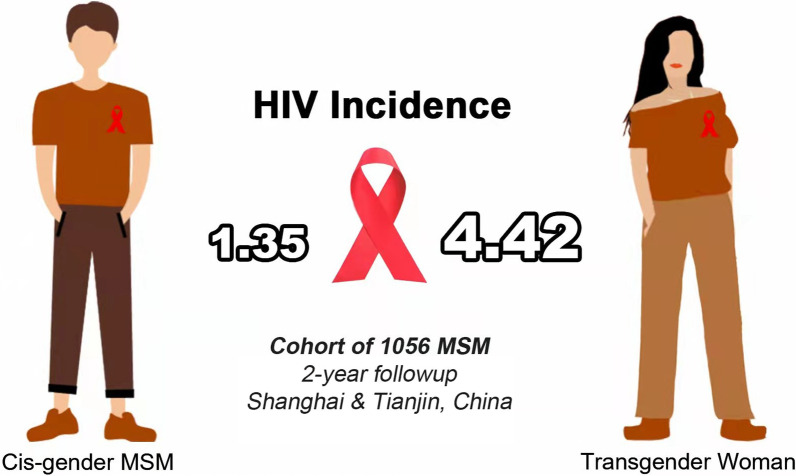

**Supplementary Information:**

The online version contains supplementary material available at 10.1186/s40249-022-00947-3.

## Background

The term “transgender” refers to a diverse population of people whose gender identity and/or expression does not correspond with the sex assigned to them at birth [1]. In this article, we use the term “transgender women” to denote those designated male at birth who identify themselves as women among men who have sex with men (MSM) whether or not they have engaged in gender transition or enhancement procedures [2]. Worldwide, transgender women bear a disproportionate burden of HIV. In 2013, a meta-analysis of data from 15 countries documented a pooled HIV prevalence of 19% and a 49-fold increased odds of HIV infection than the general population [3]. A 2016 global systematic review showed that transgender women have some of the highest concentrated HIV epidemics with prevalence up to 40% [4]. Previous studies have demonstrated an HIV prevalence among this population of 13.6–29.9% in the US, 25.0% in Brazil, 5.0–24.5% in Spain, 17.6–80.0% in Portugal, 18.1% in India and 17.5% in Thailand [5–11]. A more recent study conducted in eight African countries showed that HIV prevalence among transgender women (25%) was much higher than that in cisgender (non-transgender) MSM (cis-MSM) (14%). And compared with cis-MSM, transgender women were 2.2 times more likely to be infected with HIV [12]. A range of factors may contribute to increased risk of HIV infection among transgender women including the effects of social stigma and discrimination, the preference to be the receptive partner in anal sex to affirm their gender identity, the involvement in commercial sex, and other risky behaviors [13, 14].

China’s HIV epidemic accounts for 3% of the global HIV incidence [15]. As of October 2020, the number of people living with HIV in China exceeded 1 million for the first time. In the past ten years, MSM has become the population with the fastest growing HIV infection rates in China. Data from Chinese HIV surveillance system showed that HIV prevalence among MSM fluctuated around 8% in recent 5 years [16], which has been far higher than that of other high-risk groups. As “precise HIV prevention” strategy was more and more emphasized by Chinese government, it is imperative to analyse HIV epidemic among MSM in-depth. Transgender women, which may have relatively heightened risks of HIV infection and transmission, have long been subsumed under MSM, or as a subgroup within MSM in Chinese epidemiological studies, making their risks invisible. As transgender women have not become a part of national surveillance system, the reported HIV prevalence on transgenders to UNAIDS lack of data from China.

Nowadays, epidemiologists and researchers began to pay attention to this under-served and under-studied group in China in view of its potential role as a potential “bridge” of HIV transmission between homosexual and heterosexual populations [2]. However, as transgender women are often hidden and their gender identity data usually could not be collected completely or consistently, current related research is still limited. And despite three studies reporting a prevalence of 7.6%–14.8% among Chinese transgender women [2, 17, 18], they were primarily cross-sectional design, either with small sample size or provide little information on their differences within the broad category “MSM”. It is known that HIV incidence could well reflect HIV epidemic trend as well as the effect of HIV prevention and control efforts. And also, data on characterizing HIV incidence and risk factors among transgender women is important to help understand what role it has played in driving HIV epidemic in China and better inform future intervention strategies. Previous cohort studies in China have revealed that HIV incidence among MSM was between 3.1% and 15.6% [19–23], but none of them tried to understand heterogeneity of the MSM umbrella term or considered HIV incidence rates of transgender women, making their risks invisible. In fact, this aggregation of transgenders women into the category of general MSM may obscure understanding of how transgender women acquire HIV and fuel HIV epidemic in China.

Up to now, there is virtually no data available on HIV incidence rates of transgender women population in China. Without research on HIV incidence and risk factors, it is hard to know how different key populations contribute to Chinese HIV epidemic. To fill this gap, we conducted a prospective cohort study in two Chinese cities between January 2016 and June 2018. The objective of this study was to estimate HIV incidence among transgender women within MSM, identify and distinguish sociodemographic and sexual behavioral risk factors of HIV acquisition among transgender women from that among cis-MSM. We expect that our study will better inform evidence-based HIV intervention among transgender women in China.

## Methods

### Study design and population

We conducted a prospective open cohort study among Chinese MSM, including MSM who identify as transgender from January 2016 to June 2018 in Shanghai and Tianjin, China. To be specific, the participants were initially recruited from January to June, 2016, and were followed up until June 2018, with a frequency of approximately 6 months per visit. As the follow-up interval of some respondents was longer than 6 months, we report data up to June 2018. Participants were censored at HIV seroconversion or June 30, 2018, whichever came first.

The eligible criteria for the cohort entry were: (1) age 18 years and older; (2) being assigned male sex at birth; (3) having had anal sex with a male partner in the prior 6 months; (4) HIV negative at baseline; (5) were willing to provide written informed consent to attend the follow-up survey. Among them, “transgender women” were defined as the participants who self-identified their current gender identity does not correspond with their male sex at birth. “Cis-MSM” refers to those who reported a gender identity of male among MSM.

### Study site

This study was conducted in Shanghai and Tianjin where both local CDCs have preliminary work experience in HIV prevention programs among MSM in China, and both local CBOs demonstrated strong capacity in reaching transgender women. Baseline and follow-up visits were completed in Shanghai CSW (Commercial Sex Workers) & MSM Centre and Tianjin Shenlan Public Health Consulting Service Center, both of which focused on health education and promotion among Lesbians, Gays, Bisexuals, Transgender (LGBT) community. The basic situation of the two CBOs and their roles in HIV prevention were described in our previous cross-sectional study [2].

### Recruitment method

Local CBOs recruited participants through a wide range of methods, with a focus on finding and mobilizing transgender women to participate in this survey as much as possible. The methods included: (1) snowball sampling by contacting key informants to encourage potential subjects to participate in the study, and by word of mouth; (2) on-site mobilization in entertainment venues and urban fringes of two cities, which often gathered targeted groups; (3) mobilization on MSM-related websites; (4) widely use of internet applications such as QQ (Version 8.9, Tencent, Shenzhen, China), WeChat (Version 6.7.3, Tencent, Shenzhen, China), and Blued (Version 6.6.3, Blue city, Beijing, China), to send messages on HIV prevention and encourage them to participate. Participants were given CNY 80 (approximately USD 13) for each completed survey as compensation for transportation and meals. Subjects could make appointment by phone calls, online registration, and in-person visits, meanwhile, CBOs staff will also call participants to remind them of the next follow-up. Totally, 1545 MSM were recruited, among which 515 were identified as transgender women.

### Data collection

At each visit (i.e., baseline, first follow-up, second follow-up and third follow-up), participants’ information on demographics, gender and sexual identity, sexual risk behaviors, illicit drug use (only for transgender women), sexually transmitted infections status, experiences of stigma as well as HIV status were collected by local CBOs using a structured questionnaire. Two local CDCs were responsible for quality control of the collected data. Participants with positive HIV tests at baseline and during follow-up were referred to local CDCs to assess for treatment eligibility as well as recommendations for enrollment in local free treatment programs. All participants were provided with general information on HIV prevention in pre- and post-test counseling during the visits.

### HIV testing

The two CBOs provided HIV rapid tests with technical support from local CDCs. The specific procedure was shown in the previous study [2].

### Statistical analysis

Epidata version 3.1 (EpiData Association, Odense, Denmark) was used for data storage. SAS version 9.4 (SAS Institute, Gar, NC, USA) was used for data analysis. Demographic characteristics among transgender women and cis-MSM who entered the cohort were compared by Pearson’s *χ*^2^ tests. HIV incidence was calculated as the number of HIV seroconversions divided by the total number of person-years (PYs) of follow-up among those tested HIV-negative at baseline. Ninety-five percent confidence intervals (95% *CI*) were calculated using the exact Poisson method. Cumulative incidence of HIV infection were estimated by Kaplan–Meier methods. The follow-up time for each participant was from baseline to the date of HIV acquisition or to the date of check-in visit if HIV status was still negative. For participants with seroconversion, the follow-up time was the time interval between the first and last negative date, plus half of the time interval between the last negative date and the first positive date [24]. The risk factors of HIV acquisition were assessed by univariate and multivariate Cox regression models with time-dependent variables. We explored factors associated with HIV seroconversion in separate multivariate models for transgender women and cis-MSM. Variables that were found to be either significantly or marginally (*P* < 0.10) associated with time to seroconversion in the univariate analysis were considered for inclusion in the multivariate Cox regression models. Hazard ratios (*HR*) and 95% *CI* for the risk factors were also calculated. All testing was two-sided with significance determined at *P* ≤ 0.05.

## Results

### Enrollment and baseline characteristics

**Fig. 1:**
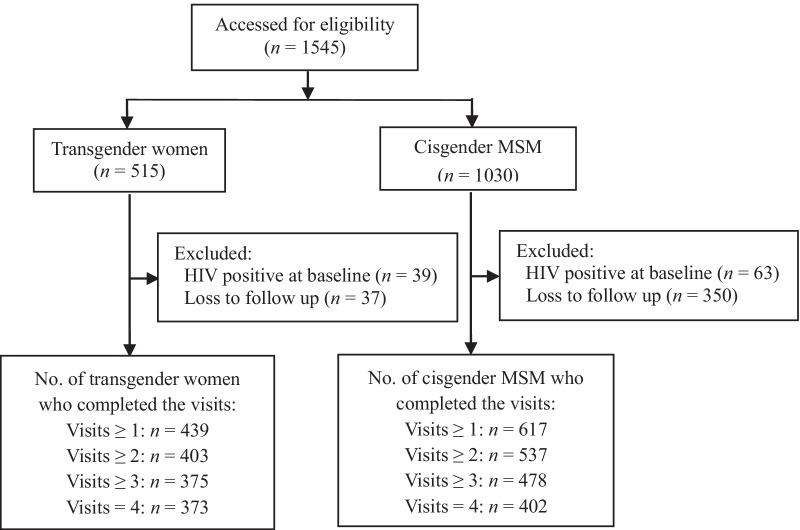
Study profile

From January 2016 to June 2018, a total of 1,545 MSM were recruited in this study, among which 515 were identified as transgender women and 1030 were cis-MSM. There were 102 HIV-positive diagnosed at the time of enrollment. After excluding participants who were HIV positive at baseline and lost to follow-up, 1056 remained in the cohort, and the overall retention rate was 68.3% (1056/1545), with 85.2% (439/515) in transgender women and 59.9% (617/1030) in cis-MSM. Totally, 89.0% (940/1056) were retained at the first follow-up, 80.8% (853/1056) at the second follow-up, and 73.4% (775/1056) at the third follow-up (Fig. 1). Among 489 who were not included in the cohort, 342 (69.9%) were from Shanghai and 315 (64.4%) lived locally ≤ 2 years. Except “city” and “local residence time”, there were no significant differences of demographic characteristics between those who were lost to follow-up and those retained in the cohort (*P* > 0.05) (See Additional file 1: Table S1).

Demographic characteristics, stratified by population type, were displayed in Table 1.

**Table 1:** Demographic characteristics of study participants at the time of enrollment (*n* = 1056)

Variables	Transgender women (*n* = 439)		Cisgender MSM (*n* = 617)		*χ*^2^	*P*
	*n*	%	*n*	%		
City					6.30	0.01
Shanghai	193	44.0	224	36.3		
Tianjin	246	56.0	393	63.7		
Age (years)					3.50	0.17
≤ 24	126	28.7	147	23.8		
25–29	150	34.2	235	38.1		
≥ 30	163	37.1	235	38.1		
Mean ± *SD*	29.4 ± 8.5		30.3 ± 8.7			
Local residence time (years)					3.26	0.07
> 2	265	60.4	410	66.5		
≤ 2	174	39.6	207	33.5		
Marital status					9.82	< 0.01
Single	330	75.2	409	66.3		
Married/cohabiting	59	13.4	118	19.1		
Divorced/widowed	50	11.4	90	14.6		
Education (years)					27.73	< 0.001
≤ 9	55	12.5	35	5.7		
10–12	128	29.2	134	21.7		
≥ 13	256	58.3	448	72.6		
Work status					58.61	< 0.001
Unemployed	54	12.3	18	2.9		
Student	31	7.1	72	11.7		
Full-time job	172	39.2	178	28.8		
Part-time job	182	41.5	349	56.6		
Average monthly income (RMB)					0.015	0.90
< 5000	294	67.0	411	66.6		
≥ 5000	145	33.0	206	33.4		
Age of sexual debut (years)					73.99	< 0.001
< 18	156	35.5	81	13.1		
≥ 18	283	64.5	536	86.9		
Mean ± *SD*	19.6 ± 3.9		20.8 ± 4.1			
Sexual orientation					2.53	0.28
Heterosexual & others	18	4.1	44	7.1		
Bisexual	99	22.6	12	1.9		
Gay	322	73.3	561	90.9		

*SD*, Standard deviation; *MSM*, Men who have sex with men

Totally, over half of the participants were from Tianjin, and nearly three-quarters were over 24 years, with the mean age 29.9 ± 8.6 years. Most participants lived locally > 2 years (63.9%), single (70.0%), with ≥ 13 years’ education (66.7%), and worked full time or part time (83.4%). There were 66.8% who reported a monthly income less than CNY 5000 (equivalent to USD 797). As presented in Table 1, transgender women were more from Shanghai (*P* = 0.01), being single (*P* = 0.01), had less schooling (*P* < 0.001), more unemployment (*P* < 0.001), more reported first sex < 18 years old, and self-identified as bisexual (*P* < 0.001). In addition, among 439 transgender women, 260 (59.2%) preferred feminine dress, 15 (3.4%) had transsexual operation and use of feminizing hormones was reported by 51 (11.3%) of them (data not shown).

### HIV risk behaviors

**Fig. 2:**
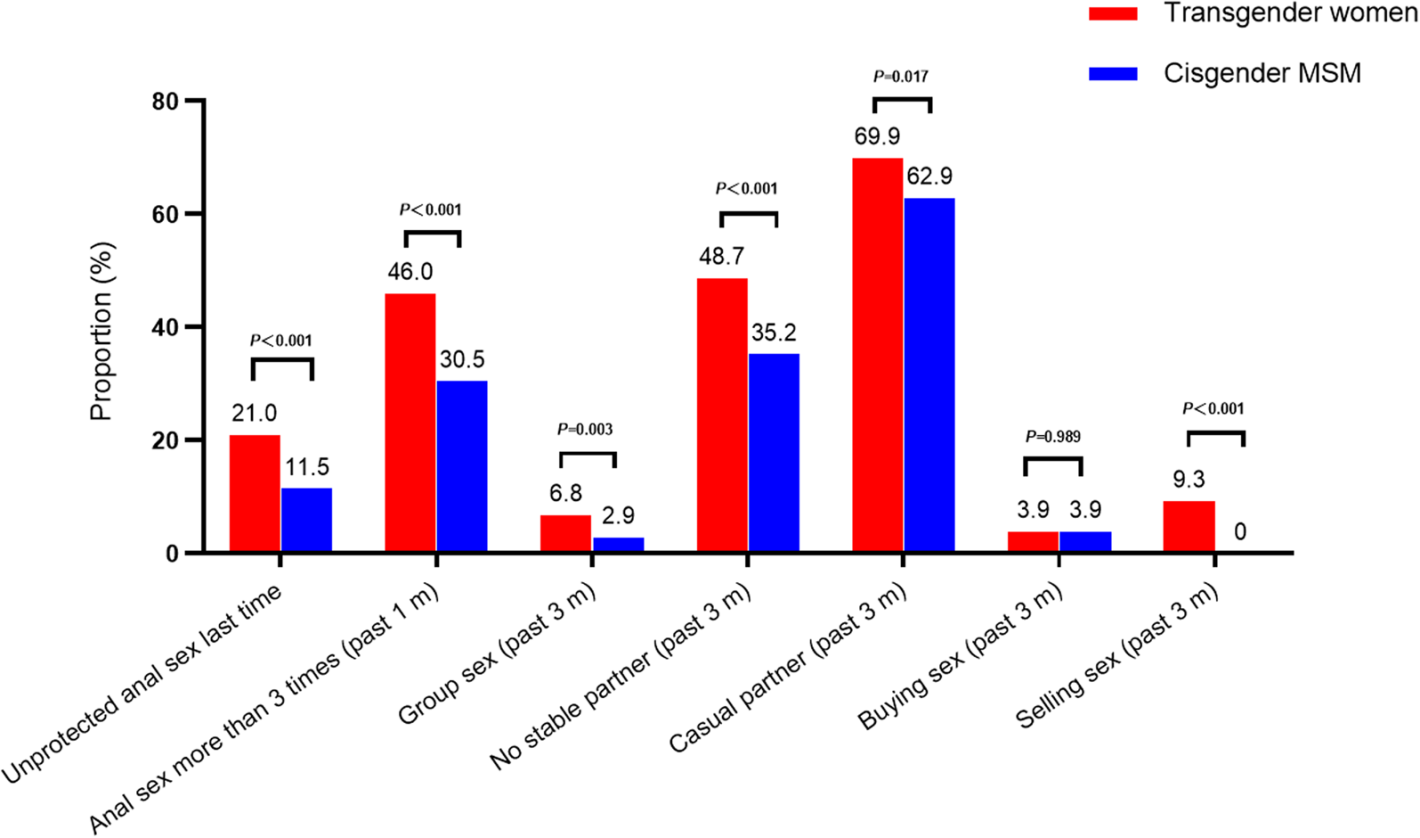
Comparison of sexual behaviors among transgender women and cisgender MSM

As shown in Fig. 2, there were several sexual behavioral differences between the two populations. Among transgender women, 21.0% reported to engage in unprotected (condomless) anal sex last time, whereas it was 11.5% in cis-MSM (*P* < 0.001). When comparing frequency of anal sex between two groups, the proportion of anal sex ≥ 3 times in the past one month was also higher in transgender women (46.0%) than that in cis-MSM (30.5%) (*P* < 0.001). Compared with cis-MSM, transgender women reported more group sex (6.8%), without stable partners (48.7%) and more casual partners (69.9%) in the past three months (*P* < 0.05). The proportion of buying sex in the past 3 months among transgender women and cis-MSM was the same, both of which were 3.9%. However, the proportion of selling sex among transgender women was 9.3%, which was significantly higher than cis-MSM (0.0%) (*P* < 0.001). The proportion of reporting discrimination-related experience was also higher (70.2%, 308/439) than that in cis-MSM (56.2%, 347/617) (*P* < 0.001). Nearly a half (44%, 193/439) of transgender women used illicit drugs in the past 6 months, including amyl nitrite (96.9%, 187/193), 5-MethoxyN,N-Diisopropyltryptamine (24.4%, 47/193), methamphetamine (10.4%, 20/193) and sildenafil (4.7%/193). In addition, there was no statistical differences of self-reported sexually transmitted infections in the past 6 months among transgender women (7.1%, 31/439) and cis-MSM (5.5%, 34/617) (*P* = 0.3).

### HIV incidence and risk factors among transgender women and cis-MSM

**Fig. 3:**
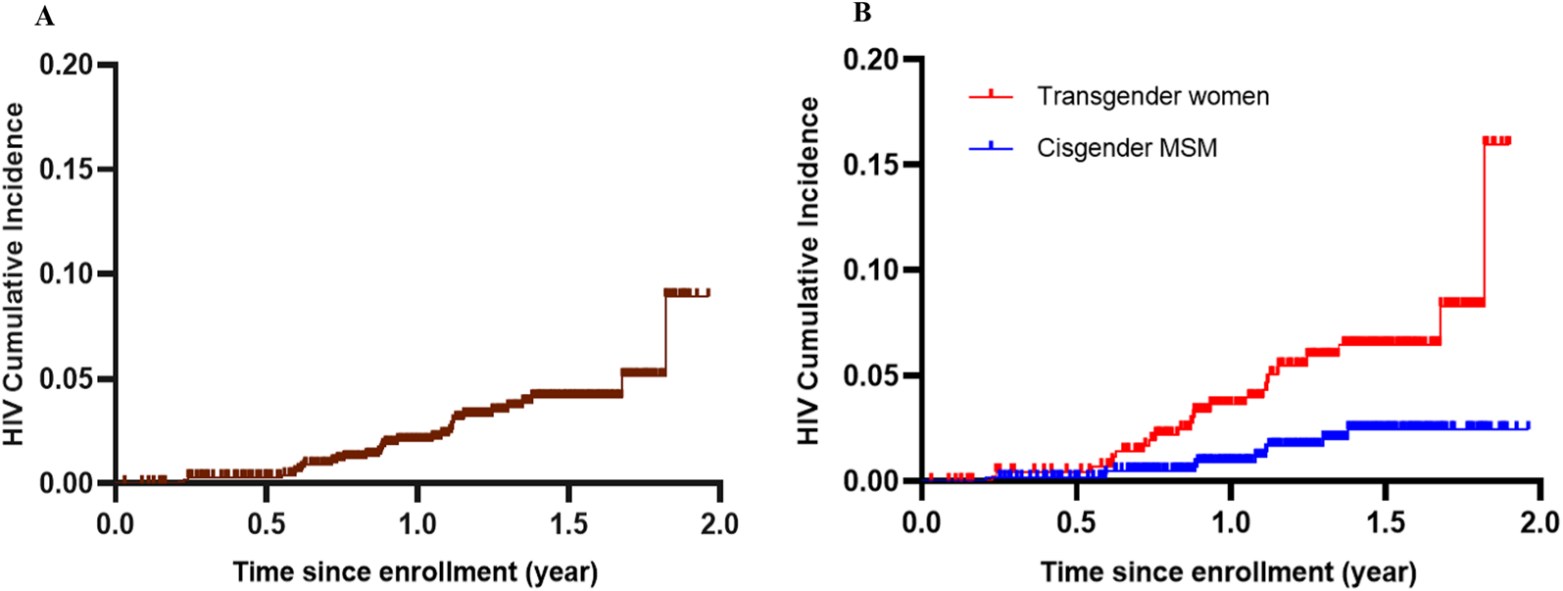
**A** Kaplan–Meier cumulative incidence among total population. **B** Kaplan–Meier cumulative incidence among transgender women and cisgender MSM

A total of 1056 participants contributed 1260.53 PYs of follow-up. A total of 33 HIV seroconversion events were observed during the follow-up period, yielding an estimated HIV incidence of 2.62 (95% *CI* 1.80–3.68) per 100 PYs. Nine seroconversions happened during the first follow-up, eight during the second follow-up, and sixteen during the third follow-up (data not shown). The incidence of HIV among transgender women was 4.42 (95% *CI* 2.80–6.62) per 100 PYs, whereas it was 1.35 (95% *CI* 0.65–2.48) per 100 PYs among cis-MSM (log-rank test *P* = 0.001). See Fig. 3-A and B.

Table 2 presents the univariate and multivariate results of the Cox regression analysis of sociodemographic and behavioral variables and their association with time to HIV seroconversion. At univariate analysis, factors significantly associated with increased risk of HIV acquisition were being transgender women, ≤ 24 years old (compared with 25–29 age group), lived locally ≤ 2 years, self-identified as bisexual, frequency of anal sex ≥ 3 times in the past one month, unprotected anal sex last time, and discrimination-related experience at all settings. After adjusting for variables including population type, age, local residence time, average monthly income, sex orientation, frequency of anal sex, unprotected anal sex, group sex, and discrimination-related experience, the multivariate analysis indicated that being transgender women [adjusted hazard ratio (a*HR*) = 3.06, 95% *CI* 1.46–6.45] and unprotected anal sex last time (a*HR* = 5.53, 95% *CI* 2.78–10.99) were independently associated with HIV incidence (Table 2).

**Table 2:** Demographic and behavioral factors associated with HIV acquisition among participants in two Chinese cities

Factors	*n*	Person-years	HIV positive (*n*)	HIV rate per 100 person-years (95% *CI*)	*HR* (95% *CI*)	*P*	a*HR* (95% *CI*)	*P*
City								
Tianjin	639	763.31	15	1.97 (1.1–3.24)	1			
Shanghai	417	497.27	18	3.62 (2.15–5.73)	1.77 (0.89–3.52)	0.10		
Population type								
Transgender women	439	519.92	23	4.42 (2.8–6.62)	3.27 (1.55–6.87)	0.00	3.06 (1.46, 6.45)	0.003
Cisgender MSM	617	740.65	10	1.35 (0.65–2.48)	1		1	
Age (years)								
≤ 24	273	364.52	12	3.29 (1.7–5.73)	1			
25–29	385	456.66	5	1.09 (0.35–2.55)	0.31 (0.11–0.87)	0.03		
≥ 30	398	471	16	3.40 (1.94–5.51)	0.97 (0.46–2.04)	0.93		
Local residence time (years)								
> 2	675	800.26	14	1.75 (0.96–2.93)	1			
≤ 2	381	460.31	19	4.13 (2.47–6.44)	1.53 (1.08–2.154)	0.02		
Marital status								
Single	739	871.2	21	2.41 (1.49–3.68)	1			
Married/cohabiting	177	213.83	7	3.27 (1.32–6.73)	1.34 (0.57–3.14)	0.51		
Divorced/widowed	140	207.15	5	2.41 (0.78–5.63)	1.11 (0.42–2.94)	0.84		
Education (years)								
≤ 9	90	104.35	3	2.87 (0.59–8.39)	1			
10–12	262	316.79	9	2.84 (1.3–5.37)	1.01 (0.27–3.72)	0.99		
≥ 13	704	871.04	21	2.41 (1.49–3.68)	0.84 (0.25–2.81)	0.77		
Work status								
Unemployed	72	84.74	2	2.36 (0.28–8.5)	1			
Student	103	130.71	3	2.30 (0.47–6.7)	0.91 (0.15–5.45)	0.92		
Full-time job	350	463.8	16	3.45 (1.97–5.6)	1.46 (0.34–6.36)	0.62		
Part-time job	531	612.93	12	1.96 (1.01–3.41)	0.83 (0.19–3.69)	0.80		
Average monthly income (RMB)								
< 1000	293	358.39	5	1.40 (0.45–3.25)	1			
1000–5000	412	480.47	17	3.54 (2.06–5.68)	2.61 (0.96–7.07)	0.06		
> 5000	351	453.33	11	2.43 (1.21–4.35)	1.79 (0.62–5.15)	0.28		
Sex orientation								
Gay	883	1097.48	23	2.10 (1.32–3.14)	1			
Heterosexual & others	62	76.24	3	3.94 (0.81–11.48)	1.84 (0.55–6.15)	0.32		
Bisexual	111	86.85	7	8.06 (3.25–16.57)	6.03 (2.48–14.66)	0.00		
Age of sexual debut (years)								
< 18	237	299.47	6	2.00 (0.73–4.34)	0.69 (0.28–1.66)	0.41		
≥ 18	819	992.71	27	2.72 (1.79–3.96)	1			
Frequency of anal sex (past 1 m)^a^								
< 3	666	826.3	14	1.69 (0.93–2.83)	1			
≥ 3	390	465.89	19	4.08 (2.44–6.36)	2.28 (1.14–4.55)	0.02		
Unprotected anal sex last time^a^								
Yes	163	182.84	16	8.75 (4.99–14.21)	5.79 (2.92–11.47)	0.00	5.53 (2.78, 10.99)	0.000
No	893	1077.73	17	1.58 (0.92–2.53)	1		1	
Group sex (past 3 m)^a^								
Yes	48	59.02	4	6.78 (1.86–17.34)	1			
No	1008	1233.16	29	2.35 (1.57–3.38)	0.37 (0.13–1.06)	0.06		
Stable partner (past 3 m)^a^								
Yes	512	649.75	15	2.31 (1.29–3.81)	1			
No	544	642.43	18	2.80 (1.66–4.43)	1.20 (0.6–2.38)	0.61		
Casual partner (past 3 m)^a^								
Yes	695	835.72	23	2.75 (1.74–4.12)	1			
No	361	456.46	10	2.19 (1.05–4.02)	0.81 (0.39–1.71)	0.58		
Self-reported sexually transmitted infections (past 6 m)^a^								
No	991	1178.87	30	3.03 (1.72–3.64)	1			
Yes	65	81.71	3	4.62 (0.75, 10.71)	1.39 (0.42–4.55)	0.51		
Discrimination-related experience at all settings (past 6 m)^a^								
Yes	655	776.8	26	3.35 (2.18–4.91)	2.68 (1.16–6.17)	0.02		
No	401	515.38	7	1.36 (0.55–2.79)	1			

*HR*, Hazard ratio; a*HR*, Adjusted hazard ratio; *CI*, Confidence interval; ^a^Time-dependent variables

As the population type was significantly associated with HIV incidence in the previous multivariate regression model for all participants, predictors of HIV acquisition among transgender women and cis-MSM were further examined in Table 3. For transgender women, being located in Shanghai, lived locally ≤ 2 years, self-identified as bisexual, and unprotected anal sex last time were positively associated with HIV acquisition at univariate analysis. After adjusting for variables including city, local residence time, sex orientation, age of sexual debut, and unprotected anal sex, the multivariate regression revealed that transgender women lived locally ≤ 2 years (a*HR* = 1.76, 95% *CI* 1.13–2.76), and unprotected anal sex last time (a*HR* = 4.22, 95% *CI* 1.82–9.79) were more likely to acquire HIV. For cis-MSM, after adjusting our multivariate analysis for variables including education, sex orientation, total frequency of anal sex, and unprotected anal sex, two factors that remained significantly associated with increased risk of HIV acquisition were frequency of anal sex ≥ 3 times in the past one month (a*HR* = 4.19, 95% *CI* 1.06–16.47) and unprotected anal sex last time (a*HR* = 5.33, 95% *CI* 1.52–18.73).

**Table 3:** Predictors of HIV acquisition among transgender women and cisgender MSM in two Chinese cities

Factors	*n*	Person-years	HIV positive (*n*)	HIV rate per 100 person-years (95% *CI*)	HR (95% *CI*)	*P*	AHR (95% *CI*)	*P*
Transgender women (*n* = 439)								
City								
Tianjin	246	333.1	8	2.40 (1.04–4.73)	1			
Shanghai	193	186.83	15	8.03 (4.48–13.25)	2.05 (1.32–3.18)	0.00		
Local residence time (years)								
> 2	265	320.6	7	2.18 (0.88–4.49)	1		1	
≤ 2	174	199.33	16	8.03 (4.58–13.03)	1.93 (1.24–3.01)	0.00	1.76 (1.13, 2.76)	0.013
Sex orientation								
Gay	322	425.43	16	3.76 (2.15–6.11)	1			
Heterosexual & others	18	20.88	1	4.79 (0.12–26.49)	1.21 (0.16–9.34)	0.86		
Bisexual	99	73.61	6	8.15 (2.99–17.67)	3.71 (1.33–10.35)	0.01		
Age of sexual debut (years)								
< 18	156	205.63	5	2.43 (0.78–5.67)	1			
≥ 18	283	314.3	18	5.73 (3.4–9.06)	2.52 (0.93–6.8)	0.07		
Unprotected anal sex last time^a^								
Yes	92	90.9	11	12.1 (6.01–21.69)	5.00 (2.18–11.49)	0.00	4.22 (1.82, 9.79)	0.001
No	347	429.03	12	2.80 (1.44–4.87)	1		1	
Buying sex (past 3 months)^a^								
Yes	17	21.2	2	9.43 (1.14–33.99)	0.44 (0.1–1.88)	0.27		
No	422	498.72	21	4.21 (2.6–6.43)	1			
Selling sex (past 3 months)^a^								
Yes	41	52.55	2	3.81 (0.46–13.71)	1			
No	398	467.37	21	4.49 (2.78–6.86)	1.3 (0.3–5.57)	0.73		
Illicit drug use (past 6 months)^a^								
Yes	193	219.85	12	5.46 (2.82–9.5)	1.57 (0.69–3.57)	0.28		
No	246	300.08	11	3.67 (1.82–6.57)	1			
Discrimination-related experience at all settings (past 6 m)^a^								
Yes	308	349.76	18	5.15 (3.05–8.14)	1			
No	131	170.16	5	2.94 (0.95–6.85)	0.54 (0.2–1.45)	0.22		
Cisgender MSM (*n* = 617)								
Education (years)								
≤ 9	35	40.49	3	7.41 (1.52–21.62)	7.51 (1.87–30.15)	0.00		
10–12	134	161.53	1	0.62 (0.02–3.42)	0.58 (0.07–4.81)	0.61		
≥ 13	448	538.63	6	1.11 (0.41–2.42)	1			
Sex orientation								
Gay	561	672.06	7	1.04 (0.42–2.14)	1			
Heterosexual & others	44	55.36	2	3.61 (0.43–13.02)	3.39 (0.7–16.34)	0.13		
Bisexual	12	13.24	1	7.55 (0.19–41.78)	8.01 (0.98–65.32)	0.05		
Frequency of anal sex (past 1 m)^a^								
< 3	429	514.4	3	0.58 (0.12–1.7)	1		1	
≥ 3	188	226.25	7	3.09 (1.25–6.36)	5.21 (1.35–20.17)	0.02	4.19 (1.06–16.47)	0.04
Unprotected anal sex last time^a^								
Yes	71	91.95	5	5.44 (1.75–12.68)	6.7 (1.94–23.19)	0.00	5.33 (1.52–18.73)	0.01
No	546	648.7	5	0.77 (0.25–1.8)	1		1	

*HR*, Hazard ratio; a*HR*, Adjusted hazard ratio; *CI*, Confidence interval; ^a^Time-dependent variables

## Discussion

This is the first prospective cohort study to evaluate HIV incidence among Chinese transgender women within MSM. Our findings showed that being transgender women and unprotected anal sex last time were related to higher HIV incidence. The incidence of HIV among transgender women in two Chinese cities was 4.42 per 100 PYs, demonstrating a threefold higher odds of HIV infection than cis-MSM. HIV incidence among transgender women in this study is moderately lower than the the pooled HIV incidence rate among Chinese MSM of 5.0–5.5 PYs in the systematic review and meta-analysis in 2015 and 2016 [25, 26], however, it is higher than that in general MSM in Beijing during 2017–2018 (3.09 per 100 PYs) [27] and general MSM in Chengdu in 2018 (3.47 per PYs) [28]. And it is higher than the HIV incidence among transgender women of 2.9/100 per PYs in New York, US, 2.3 per 100 PYs in Lima, Peru, and 3.6 per 100 PYs in 11 randomised controlled trial sites in Brazil, Ecuador, Peru, South Africa, Thailand, and the USA in 2014 [29–31]. In general, our results were consistent with the fact that compared to cis-MSM population, transgender women were at higher risk of becoming infected with HIV in other countries. Meanwhile, it also contribute important nuance to understanding HIV epidemic in China, where HIV surveillance and research mainly considered binary gender these years.

In terms of loss to follow-up, an open cohort analysis in Thailand showed that during 3-year follow-up, 1411 participants contributed 986.5 PYs [32]; another open cohort study conducted in Tianjin, Harbin, Chongqing, Nanjing and Xi'an of China demonstrated that 4305 MSM contributed 4678.4 PYs in two and half years’ follow-up [24]; and in a more recent open cohort in Beijing, 1065 PYs were observed among 1937 person-years in one and half years [27]. In our study, 1056 MSM contributed 1260.53 PYs during the 2-year observation period, and it is worth mentioning that it’s not easy to maintain the follow-up visits in this open chort, especially for transgender women as a marginalized and hard-to-reach population.

In our study, transgender women were more likely to report being single, less schooling, more unemployment, and more self-identified as bisexual compared with cis-MSM. Very few transgender women took feminizing hormones or used surgical procedures to affirm their identity this study, while hormone use among transgender women was reported to be 27–93% in the US, Thailand and Canada [6, 33, 34]. It should be noted that access to appropriate therapies is still very difficult in China, and the majority of transgender women could not complete the “transition”, which may contribute to low self-esteem and poor mental health, and thereby increase the likelihood of risky behaviors [35]. There was hardly any policies or regulations that help transgenders to access medical, legal, and social recognition and services. It should also be noted that this is a group who disrupt the gender binary in which Chinese culture is grounded. Moreover, there was still no practice guide for clinicians on providing transgender medical care, and the cost of gender-affirmative procedures can be quite high, which may further hinder their access to medical services. In our study, transgender women were more likely to have no stable partners than cis-MSM. It was inferred that lack of intimacy in the case of “no regular partners” may be unfavourable for their gender transition, and they would probably try to satisfy their psychological needs by pursuing condomless sex. Synchronized with this were the relatively higher proportion of casual sex partners, condomless anal sex and engagement in group sex among transgender women in our study. The proportion of selling sex was also higher among transgender MSM as compared to cis-MSM. Study results suggest that non-dominant social identity contributes to their social exclusion, which in turn makes them unable to participate in societal mainstream activities, and pushes them towards entering the risky business of selling sex [9, 36]. Moreover, transgender participants were more likely to report experiences of discrimination than cis-MSM in this study. As such, these factors mentioned above including limited access to gender-affirming, low self-esteem, experience of discrimination, and engagement in selling sex tend to link and interact with each other [13], leading to sexual risky behaviors.

Age was not found to be a predictor of HIV incidence among two groups in this study. But in other studies young age has been associated with increased risk of HIV acquisition [37, 38]. This may be because that our cohort with an average age of 29.9 was overall younger compared with other studies, and differences were not found. In spite of this, we still emphasize early HIV prevention, especially among transgender women, in consideration of their relatively high incidence of HIV. Although sex orientation was only of statistical significance in univariate analysis among two populations, another study result showed that compared to gay men, the bisexual men reported more condomless anal sex with their casual partners, which may increase HIV risks [39]. In addition, we noticed that a quarter of transgender women in our study were bisexuals with HIV incidence up to 8 per 100 PYs. As transgender women may have sex with both heterosexual men and MSM, the sexual behaviors of bisexuals may add more complexity in understanding the chain of HIV infection and transmission among transgender women.

Among cis-MSM in this study, two risk factors were identified to be associated with HIV acquisition, including unprotected anal sex last time and frequency of anal sex ≥ 3 times in the past one month, which were consistent with other HIV incidence studies showing that condomless anal sex with a high frequency was deemed to be important predictors of HIV incidence [40, 41]. By referring to other relevant studies [42–44], we took 2 years as the cut-off point to differentiate those locals and migrants, to facilitate comparison with other research results. Our study revealed higher HIV incidence of those lived locally no more than 2 years among transgender women compared to those lived locally over 2 years, which is consistent with previous studies showing that compared with native populations, migrants were at increased risk of HIV attributable to individual and structural barriers that increase their vulnerability [45, 46]. Specifically, they may experience social and economic changes, loss of family support, and exposure to unfamiliar social and sexual circumstances, and may engage in unprotected anal intercourse after arrival as a form of stress relief or social connection [47]. Additionally, this observation indicated that migrant transgender women should receive more attention in future HIV epidemic surveillance and behavior intervention efforts, and in view of their high incidence, large-scale HIV screening should also be considered to prioritize for this subgroup. The results provided a starting point for further consideration of factors related to migration influencing HIV risk for this gender minority of MSM [48].

We observed that up to a half of transgender women used illicit drugs. Yet, unlike other studies of similar populations [49, 50], this study did not find a positive correlation between illicit drug use and HIV acquisition among transgender women. It should be noted that most transgender women self-reported a frequency of drug use no more than once a month, which may partially explain why illicit drug use was not significantly correlated with HIV incidence among them. Meanwhile, this result concurs several other studies in China in which no correlation between illicit drug use and HIV acquisition was found [22, 51]. Nonetheless, in consideration of many literature demonstrating illicit drug use may further aggravate HIV vulnerability of transgender women [5, 13, 52, 53], drug use should still be closely monitored among this population.

The study has several limitations. First, relatively low HIV seroconversion counts among cis-MSM may affect the HIV incidence calculations. Second, our study subjects were recruited using a non-random sampling method which could have led to selection bias. Third, responses to questions about sexual behavior and illicit drug use were self-reported, which may be subject to social desirability and recall bias. Moreover, we could not compare illicit drug use between transgender women and cis-MSM because related information was not collected among cis-MSM in our study. And information on receptive or insertive sex roles, which may also played a role in HIV infection was not collected among transgender women and cis-MSM. However, we focused on comparison of sexual risk behaviors and found major differences in demographic and sexual risk factors among transgender women and cis-MSM.

Despite the above limitations, this study was an important first step in filling the void of information on HIV incidence among transgender women in China, and helped raise the visibility of HIV epidemic among this population. In recent years, China has progressively scaled up its responses to HIV epidemic, and there is an increasing nationwide recognition of the importance of focusing HIV prevention on specific at-risk populations, especially MSM [54]. In such settings, further characterizing HIV epidemiology by gender identity within MSM is essential to China with a concentrated HIV epidemic. Our study collected such data and the results showed that HIV incidence among Chinese transgender women was high, which highlight the urgent need to understand more specific behavioral characteristics of transgender women in China, and pointed to a need for more tailored HIV prevention for this population.

Current HIV programs only target the general MSM, and programs designed for transgender women are limited. On one hand, the existing strategies and measures on HIV intervention may not be effective at reaching transgender women, on the other hand, transgender women, who may still face access barriers to social, medical and psychological gender affirmation, may not be willing to participate in current health network targeted to MSM. In view of this, future programs should pay more attention on addressing the challenge of this population to consider their unique mental health needs and ameliorate existing cultural and social stigma they face. In addition, transgender women are still a very hidden population in China, thus, intervention delivery through social media, including mobile apps may also needed [28]. In addition to traditional interventions such as health education and condom use, newer strategies such as pre-exposure prophylaxis should also consider to include transgender women [55, 56] to ensure that this population in China are not left behind.

## Conclusions

In conclusion, stronger HIV prevention strategies should be implemented to target transgender women who were migrants and those tended to engage in unprotected anal sex. Moreover, as research on risk of HIV incidence among transgender women is in its infancy, future research should focus more on vulnerabilities and risks of HIV acquisition among this population, and take a deeper look into individual and contextual factors.

## Supplementary Information

**Additional file 1: Table S1.** Demographic characteristics of participants at baseline and follow-up (*n *= 1545).

## Data Availability

Please contact the corresponding author for data requests.

## Abbreviations

AIDS: Acquired Immune Deficiency Syndrome
CBO: Community-based Organization
CDC: Center for Disease Control and Prevention
*CI*: Confidential intervals
HIV: Human immunodeficiency virus
IQR: Interquartile range
MSM: Men who have sex with men
STI: Sexually transmitted infection
cis-MSM: Cisgender men who have sex with men
PYs: Person-years
*HR*: Hazard ratio
a*HR*: Adjusted hazard ratio
*SD*: Standard deviation
