# Supplementary material for: HIV incidence and risk factors among transgender women and cisgender men who have sex with men in two cities of China: a prospective cohort study
Source: Infect Dis Poverty. 2022 Mar 7;11:26. doi: 10.1186/s40249-022-00947-3 (PMC8900389; doi:10.1186/s40249-022-00947-3)
Supplement: Supplementary file 1 — Additional file 1: Table S1. Demographic characteristics of participants at baseline and follow-up (n = 1545). [file 40249_2022_947_MOESM1_ESM.docx]

| Table S1 Demographic characteristics of participants at baseline and follow-up (n=1545) | | | | | | | |
| --- | --- | --- | --- | --- | --- | --- | --- |
| Variables | | In the Longitudinal Cohort | | Not in the Longitudinal Cohort | | χ^2^ | *P* |
|  |  | n | % | n | % |  |  |
| City | | | | | |  |  |
|  | Shanghai | 417 | 39.5 | 342 | 69.9 | 124.00 | ＜0.001 |
|  | Tianjin | 639 | 60.5 | 147 | 30.1 |  |  |
| Age (years) | | | | | | 1.98 | 0.37 |
|  | ≤24 | 273 | 25.9 | 142 | 29.0 |  |  |
|  | 25-29 | 385 | 36.5 | 165 | 33.7 |  |  |
|  | ≥30 | 398 | 37.7 | 182 | 37.2 |  |  |
| Marital status | | | | | | 1.62 | 0.44 |
|  | Single | 739 | 70.0 | 327 | 66.9 |  |  |
|  | Married/cohabiting | 177 | 16.8 | 93 | 19.0 |  |  |
|  | Divorced/widowed | 140 | 13.3 | 69 | 14.1 |  |  |
| Local residence time (years) | | | | | | 108.42 | ＜0.001 |
|  | ＞2 | 675 | 63.9 | 174 | 35.6 |  |  |
|  | ≤2 | 381 | 36.1 | 315 | 64.4 |  |  |
| Ethnicity | | | | | | 3.21 | 0.07 |
|  | Han | 1032 | 97.7 | 470 | 96.1 |  |  |
|  | Others | 24 | 2.3 | 19 | 3.9 |  |  |
| Education (years) | | | | | | 3.64 | 0.16 |
|  | ≤9 | 90 | 8.5 | 49 | 10.0 |  |  |
|  | 10-12 | 262 | 24.8 | 101 | 20.7 |  |  |
|  | ≥13 | 704 | 66.7 | 339 | 69.3 |  |  |
| Work status | | | | | | 3.73 | 0.29 |
|  | Unemployed | 72 | 6.8 | 31 | 6.3 |  |  |
|  | Student | 103 | 9.8 | 60 | 12.3 |  |  |
|  | Full-time job | 350 | 33.1 | 144 | 29.4 |  |  |
|  | Part-time job | 531 | 50.3 | 254 | 51.9 |  |  |
| Average monthly income (RMB) | | | | | | 3.39 | 0.07 |
|  | ＜5000 | 705 | 66.8 | 303 | 62.0 |  |  |
|  | ≥5000 | 351 | 33.2 | 186 | 38.0 |  |  |
